# Aging is a Risk Factor for Rheumatoid Arthritis in Rats: Therapeutic Potential of 4‑(Phenylselanyl)-2H-chromen-2-one

**DOI:** 10.1021/acsomega.5c02655

**Published:** 2025-06-09

**Authors:** Caren Aline Ramson da Fonseca, Jaini Janke Paltian, Ketlyn Pereira da Motta, Carolina Cristóvão Martins, Jean Carlo Kazmierczak, Ricardo Frederico Schumacher, Mauro Pereira Soares, Cristiane Luchese, Ethel Antunes Wilhelm

**Affiliations:** † Center of Chemical, Pharmaceutical and Food Sciences, Graduate Program in Biochemistry and Bioprospecting, Federal University of Pelotas, Pelotas City 96010-900, Brazil; ‡ Chemistry Department, Graduate Program in Chemistry, 28118 Federal University of Santa Maria, Santa Maria City 97105-900, Brazil; § Regional Diagnostic Laboratory, Faculty of Veterinary, Federal University of Pelotas, Pelotas City 96010-900, Brazil

## Abstract

The underlying mechanisms of rheumatoid arthritis (RA) remain inconclusive; nevertheless, several factors may contribute to developing and exacerbating the disease’s signs and symptoms. It is well established that the structure and function of the organism decline with age; consequently, the aging process has become a significant risk factor for several human diseases. Thus, the current study investigated aging as a risk factor for RA in rats and the potential of 4-(phenylselanyl)-2H-chromen-2-one (4-PSCO) as a new therapeutic strategy. Arthritis was induced by intraplantar injection (i.pl.) of complete Freund’s adjuvant (CFA; 0.1 mL) in the left hind paw of young and aged adult male Wistar rats. The 4-PSCO (1 mg kg^–1^) was administered via the intragastric route for 10 days. CFA administration in aged rats aggravated pain sensitivity by increasing oxidative damage in the central and peripheral nervous systems. The treatment with 4-PSCO reversed pain sensibility, reduced the inflammatory process, and restored the body weight and spleen index. Additionally, 4-PSCO reduced oxidative stress in the paw and spinal cord. Our findings highlight 4-PSCO as a promising therapeutic alternative to develop a more effective and safer drug to treat RA and underscore age-related differences as an important factor for RA pathogenesis.

## Graphical abstract

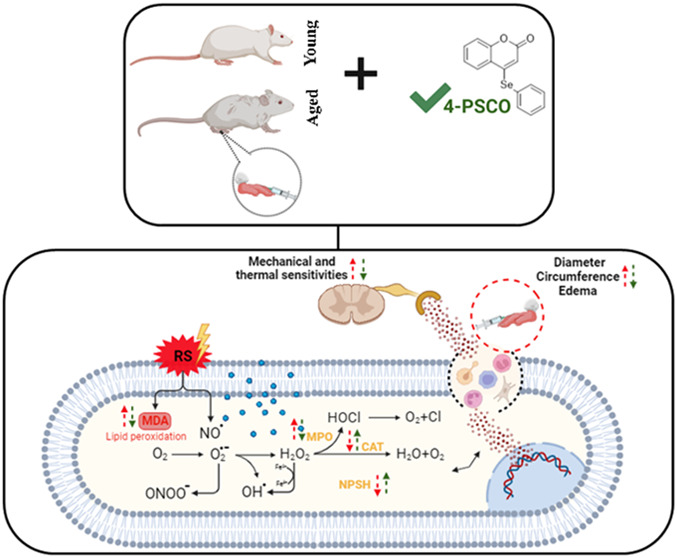

## 1 Introduction

Aging is characterized by alterations in the organism’s immunological homeostasis due to the accumulation of molecular and cellular damage.^1–3^ There is compelling evidence indicating a global increase in the population aged 60 and above, projected to rise by 22% by 2050.^4,5^ This process results in a gradual decline in physical and cognitive capacities, increased vulnerability to age-related morbidities, and ultimately, death.^2,3^ Given that aging is one of the main risk factors for chronic diseases, understanding its underlying mechanisms is essential for identifying therapeutic targets and developing effective pharmacological agents to treat age-related conditions.

In this manner, rheumatoid arthritis (RA) is a prominent autoimmune and inflammatory disease worldwide, characterized by synovial membrane inflammation and damage to cartilage and bone tissue within joints.^6^ RA affects approximately 0.5–2% of the global population, with a higher incidence among aged individuals.^7^ Clinically, RA is classified into two subsets: elderly onset RA, affecting 10–33% of individuals over 60 years, and young-onset, which occurs between the ages of 30 to 50 years.^8^

Despite extensive research, the etiology of RA remains elusive, contributing to challenges in accurate prognosis. Oxidative stress has emerged as a pivotal mechanism implicated in both the initiation and perpetuation of RA.^9^ Elevated levels of reactive species (RS) contribute to inflammatory and immunological cellular responses, promoting cartilage degeneration and acting as signaling molecules in these processes.^10,11^ Furthermore, aging exacerbates RA progression, primarily due to increased systemic inflammation and oxidative stress.^12,13^

Pain, a prevalent and debilitating symptom in RA, is closely associated with peripheral joint inflammation.^14^ Although inflammatory pain is a hallmark of RA, growing evidence suggests that neuropathic pain also plays a crucial role, further aggravating the clinical condition and complicating therapeutic management.^15–17^ Studies indicate that arthritic pain is not solely a consequence of peripheral inflammation but results from a complex interplay between central sensitization and local inflammatory mechanisms.^17–19^ This interaction involves neurochemical and immunological pathways that modulate nociception, contributing to persistent pain even during sustained remission or periods of low inflammatory activity.

Despite the availability of numerous therapeutic strategies, effective pain management remains inadequate for many patients. Variability in treatment responses and adverse effects often results in treatment discontinuation.^20,21^ These challenges underscore the need for novel therapeutic approaches that better target the underlying mechanisms of pain in RA and offer more effective and long-lasting relief.

In recent years, organoselenium compounds have emerged as promising agents in the pharmacological field, characterized by their straightforward synthesis and significant therapeutic properties. Experimental evidence suggests that these compounds have the potential to attenuate oxidative stress and effectively modulate inflammatory and nociceptive responses.^22–24^ Simultaneously, coumarins have gained recognition as privileged scaffolds in medicinal chemistry due to their structural versatility. These features have facilitated the development of numerous coumarin-based derivatives exhibiting a broad spectrum of biological activities, including antioxidant, anti-inflammatory, antinociceptive, and antimicrobial properties, as extensively documented in the literature.^25–28^

The unique chemical structure of selenium-coumarin compounds, such as 4-PSCO, has spurred growing scientific investigation. Preclinical studies have shown that combining parts with coumarin scaffolds enhances the pharmacological effects of these molecules. Padilha et al.^29^ and Arsenyan et al.^30^ reported the antioxidant properties of selenium-coumarin compounds and suggested their potential in alleviating oxidative stress linked with pain and inflammation. More recently, we investigated the effects of the compound 4-PSCO on pain-associated proteins using computational molecular docking techniques.^31^ It was demonstrated to have antinociceptive and antiedematogenic properties in acute and inflammatory pain models and no toxicity in evaluated parameters.

Given the persistent challenge of managing RA-associated pain across different life stages, this study aimed to investigate the pharmacological effects of 4-PSCO on mechanical allodynia and thermal hyperalgesia in an experimental model of RA in young adult and aged rats. Additionally, the study explored the influence of aging on oxidative stress and inflammation to demonstrate the modulatory effects of 4-PSCO on these pathological processes.

## 2 Results

### 2.1 Effects of Aging and 4-PSCO on Arthritic Scores in CFA-Induced RA

The arthritic scores in young adult and aged rats exposed to CFA are presented in Figure 1A,1B. On day 7 of the experimental protocol, young and aged control animals subjected to CFA induction exhibited elevated arthritic scores (Figure 1A,1B). Notably, RA-like symptoms persisted for 21 days following CFA induction in both age groups. On day 7, aged CFA-induced animals displayed higher arthritic scores compared to the young CFA-induced group, suggesting that aging may exacerbate the signs and symptoms of RA (Figure 1A,1B). By day 21, daily treatment with 4-PSCO effectively reduced arthritic parameters, including swelling, edema, and erythema in the ipsilateral paw of both young and aged CFA-induced rats when compared to the respective untreated groups (Figure 1A,1B).

**Figure 1:**
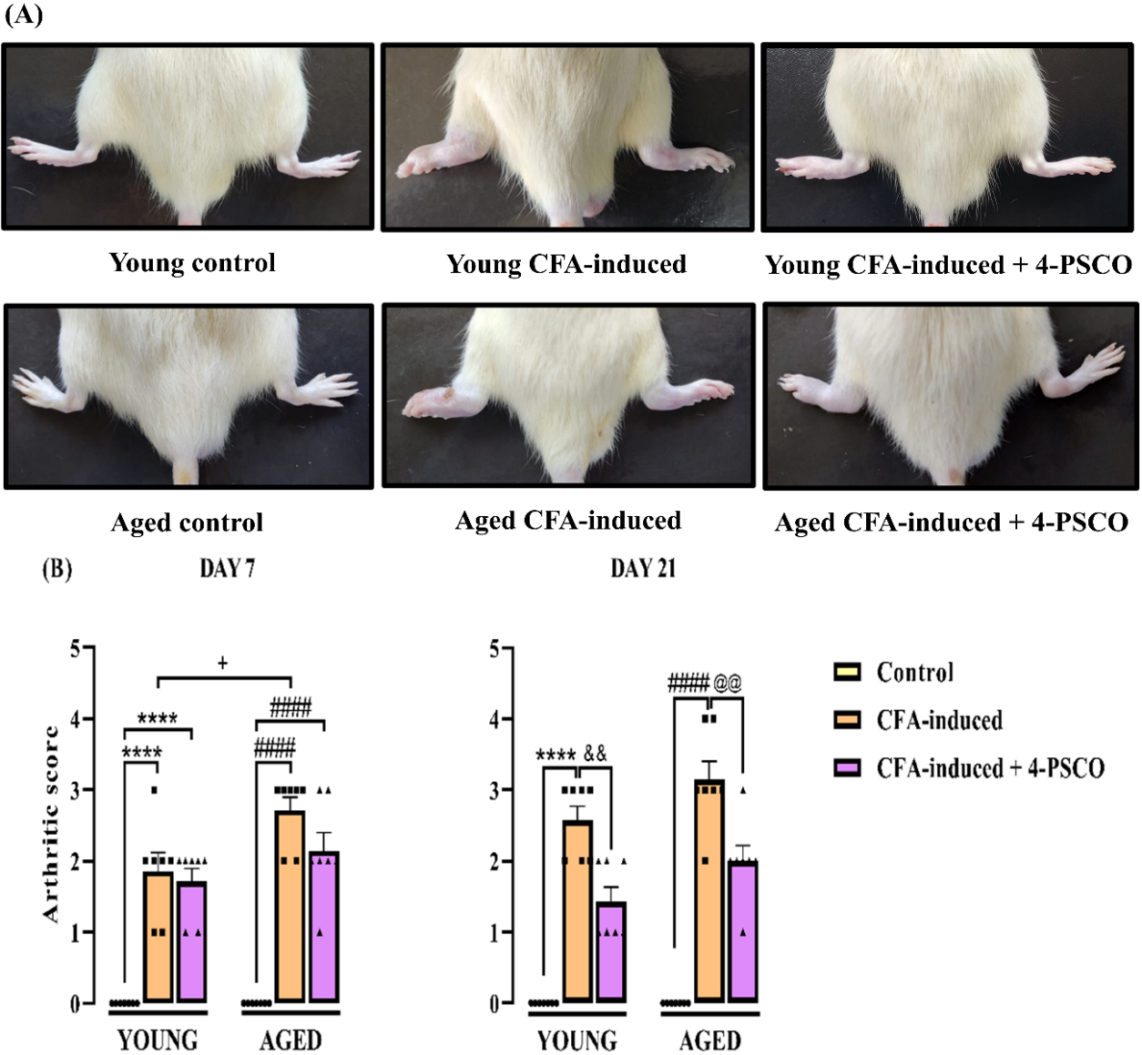
Effects of aging and 4-(phenylselanyl)-2H-chromen-2-one (4-PSCO) (1 mg kg^–1^, i.g.) on rheumatoid arthritis (RA)-like symptoms induced by Complete Freund’s Adjuvant (CFA) (0.1 mL, i.pl.). (A) hind paw images, and (B) the average score of arthritis. Each point represents the mean of 7 rats in each group. (****) *p* < 0.0001 denotes significance levels compared with the young control group; (####) *p* < 0.0001 denotes significance levels compared with the aged control group; (+) *p* < 0.05 denotes significance levels compared with the aged CFA-induced group; (&&) *p* < 0.01 denotes significance levels compared with the young CFA-induced +4-PSCO group; and (@@) *p* < 0.01 denotes significance levels compared with the aged CFA-induced +4-PSCO group (Two-way ANOVA followed by Tukey’s test).

### 2.2 Effects of Aging and 4-PSCO on Body Weight Variations in CFA-Induced RA

Due to age-related differences, baseline body weight (day 1) differed significantly among young control, aged control, young CFA-induced, and aged CFA-induced rats. Aged arthritic animals (aged CFA-induced group) exhibited significant body weight loss on days 7, 12, and 16 compared to the aged control group (Figure 2). Notably, by day 21, body weight loss in the aged CFA-induced group reached 16% (ANOVA: *F*_(2, 36)_ = 18.57, *p* < 0.0001). In contrast, the young CFA-induced group showed a significant reduction in body weight only on day 21 compared to the young group. Daily treatment with 4-PSCO effectively normalized body weight in aged CFA-exposed animals (Figure 2).

**Figure 2:**
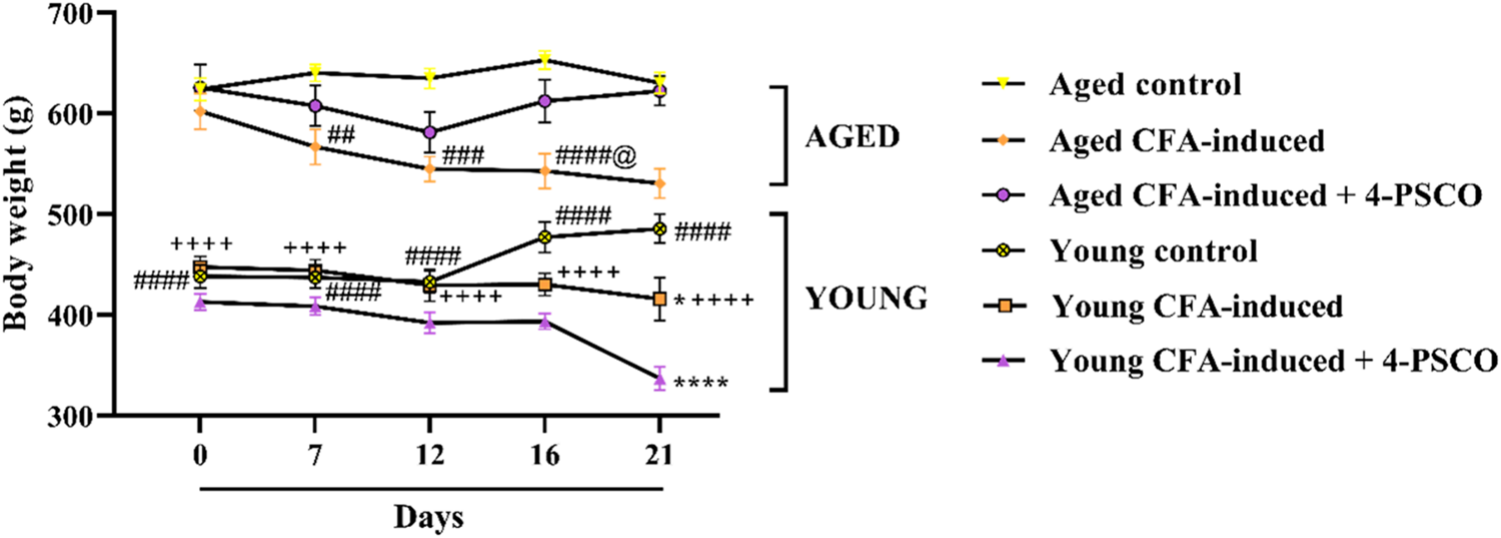
Effects of aging and 4-(phenylselanyl)-2H-chromen-2-one (4-PSCO) (1 mg kg^–1^, i.g.) on the body weight of rats with rheumatoid arthritis (RA)-like symptoms induced by Complete Freund’s Adjuvant (CFA) (0.1 mL, i.pl.). Each point represents the mean of 7 rats in each group. (*) *p* < 0.05, and (****) *p* < 0.0001 denote significance levels compared with the young control group; (##) *p* < 0.01, (###) *p* < 0.001, and (####) *p* < 0.0001 denote significance levels compared with the aged control group; (++++) *p* < 0.0001 denotes significance levels compared with the aged CFA-induced group; (@) *p* < 0.05, and (@@) *p* < 0.01 denote significance levels compared with the aged CFA-induced +4-PSCO group (Two-way ANOVA followed by Tukey’s test).

### 2.3 Suppressive Impact on RA-Like Signs by the Anti-Inflammatory Effects of 4-PSCO

As shown in Figure 3, a substantial increase in paw diameter (Figure 3A) and circumference (Figure 3B) was observed on day 7 following CFA injection in the young CFA-induced and aged CFA-induced groups compared to their respective control groups (young and aged control groups). A notable difference in paw circumference was also detected between the young CFA-induced and aged CFA-induced groups on day 7. By day 21, the young CFA-induced group exhibited increases of 38% in paw diameter and 43% in circumference. In the aged CFA-induced group, paw diameter and circumference increased by 34 and 32%, respectively, compared to the aged control group. Significantly, treatment with 4-PSCO (1 mg kg^–1^, i.g.) effectively attenuated these increases in both young and aged CFA-exposed animals, considerably reducing paw diameter (ANOVA: *F*_(2,36)_ = 67.13, *p* < 0.0001; Figure 3A) and circumference (ANOVA: *F*_(2, 36)_ = 70.55, *p* < 0.0001; Figure 3B).

**Figure 3:**
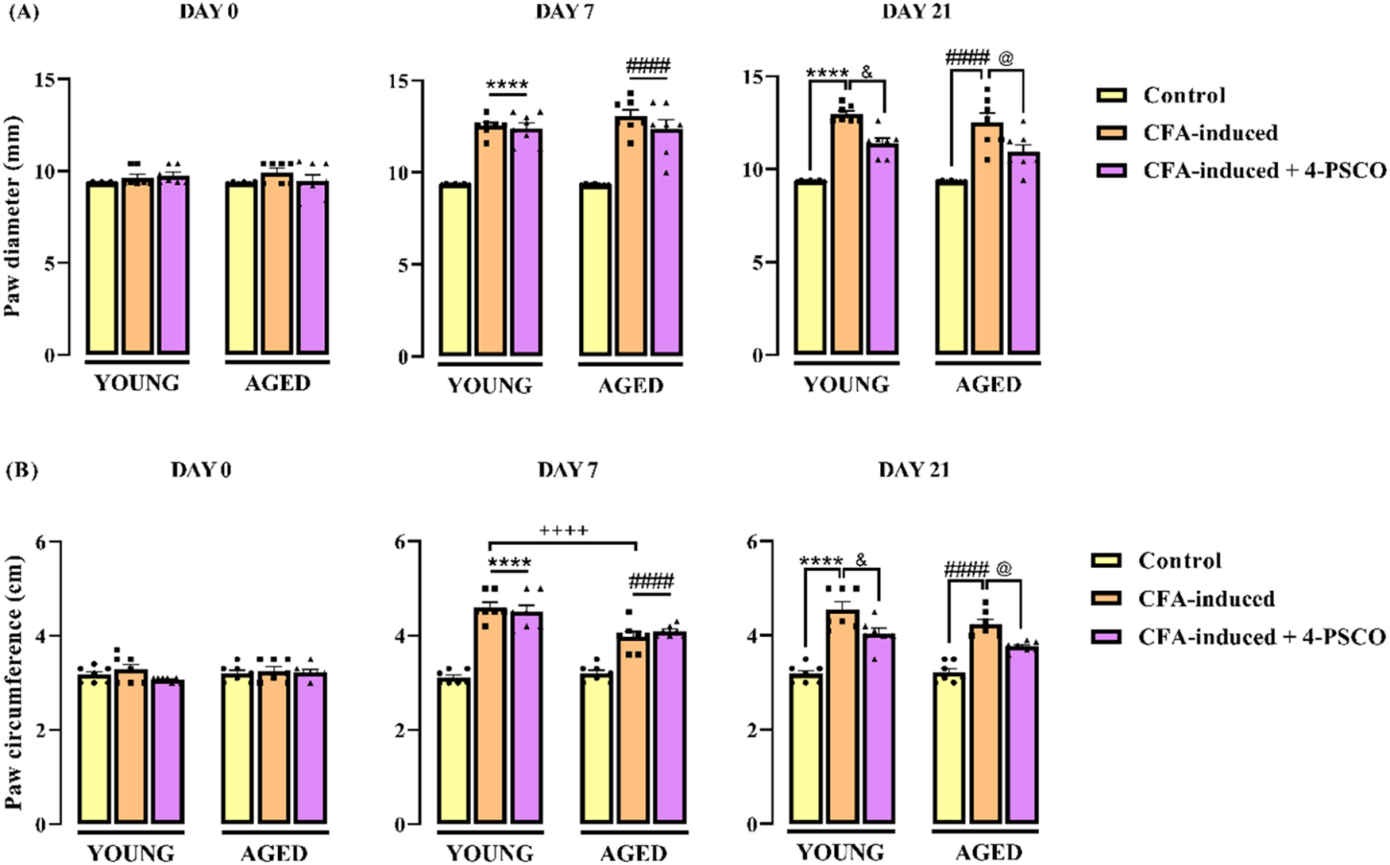
Effects of aging and 4-(phenylselanyl)-2H-chromen-2-one (4-PSCO) (1 mg kg^–1^, i.g.) on paw diameter (A) and circumference (B) of rats exposed to Complete Freund’s Adjuvant (CFA) (0.1 mL, i.pl.). Each point represents the mean of 7 rats in each group. (****) *p* < 0.0001 denotes significance levels compared with the young control group; (####) *p* < 0.0001 denotes significance levels compared with the aged control group; (++++) *p* < 0.0001 denotes significance levels compared with the aged CFA-induced group; (&) *p* < 0.05 denotes significance levels compared with the young CFA-induced +4-PSCO group; and (@) *p* < 0.05 denotes significance levels compared with the aged CFA-induced +4-PSCO group (Two-way ANOVA followed by Tukey’s test).

Similarly, young and aged rats exposed to CFA exhibited a significant increase in paw edema compared to their control groups. Statistical differences were also observed between the young and aged CFA-induced groups, whereas no significant difference was found between the young and aged controls. Notably, treatment with 4-PSCO (1 mg kg^–1^, i.g.) effectively reduced paw edema in CFA-exposed rats, with reductions of approximately 37% in young and 53% in aged animals (ANOVA: *F*_(2, 36)_ = 151.8, *p* < 0.0001) (Figure 4A).

**Figure 4:**
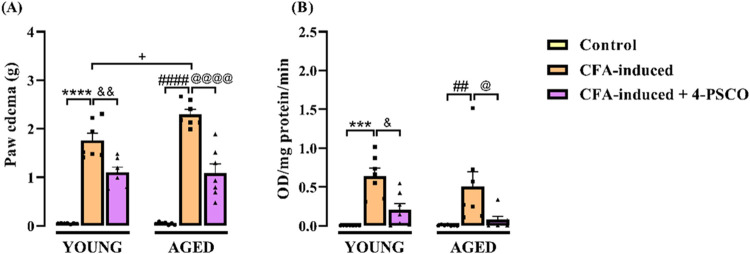
Effects of aging and 4-(phenylselanyl)-2H-chromen-2-one (4-PSCO) (1 mg kg^–1^, i.g.) on paw edema (A) and myeloperoxidase (MPO) activity (B) of rats exposed to Complete Freund’s Adjuvant (CFA) (0.1 mL, i.pl.). Each point represents the mean of 7 rats in each group. (***) *p* < 0.001 and (****) *p* < 0.0001 denote significance levels compared with the young control group; (##) *p* < 0.01 and (####) *p* < 0.0001 denote significance levels compared with the aged control group; (++++) *p* < 0.0001 denotes significance levels compared with the aged CFA-induced group; (&) *p* < 0.05, and (&&) *p* < 0.01 denote significance levels compared with the young CFA-induced +4-PSCO group; (@) *p* < 0.05, and (@@@@) *p* < 0.0001 denote significance levels compared with the aged CFA-induced +4-PSCO group (Two-way ANOVA followed by Tukey’s test).

Given the heightened inflammatory response induced by CFA, we assessed myeloperoxidase (MPO) activity. As shown in Figure 4B, CFA significantly increased MPO activity in the paw of both young and aged rats compared to their respective control groups. Aging alone did not alter MPO enzymatic activity, and no statistical difference was observed between the young CFA-induced and aged CFA-induced groups. However, treatment with 4-PSCO effectively normalized MPO activity, reducing it by approximately 68% in young and 84% in aged CFA-exposed rats (ANOVA: *F*_(2, 36)_ = 19.79, *p* < 0.0001) (Figure 4B).

Figure 5 presents the histological profiles of the rat paws following CFA-induced RA. The paws of young (Figures 5.1 and 5.4) and aged (Figures 5.7 and 5.10) control animals showed standard histological architecture. In contrast, the young and aged CFA-induced groups exhibited skin flattening due to intense edema and abundant eosinophilic inflammatory infiltrate. Marked lymphatic vessel dilatation and moderate mononuclear cell infiltration were also observed (Figures 5.2 and 5.9). In young rats, foci of eosinophilic infiltrate induced by CFA were randomly distributed (Figure 5.2). Furthermore, young and aged CFA-induced groups showed intra-articular spaces expanded by an intense eosinophilic inflammatory infiltrate. The joint surfaces were dissociated, and within the joint space, cell debris, edema, and hemorrhage were present (Figures 5.5 and 5.6). The synovial membrane was thickened by an intense eosinophilic inflammatory infiltrate associated with chondrocyte necrosis, hemorrhage, and lymphatic vessel dilatation. The interosseous and subcutaneous connective tissues were markedly expanded due to edema, fibrin, and intense eosinophilic inflammatory infiltrate. Prominent lymphatic vessel dilatation and eosinophilic perivasculitis were also evident.

**Figure 5:**
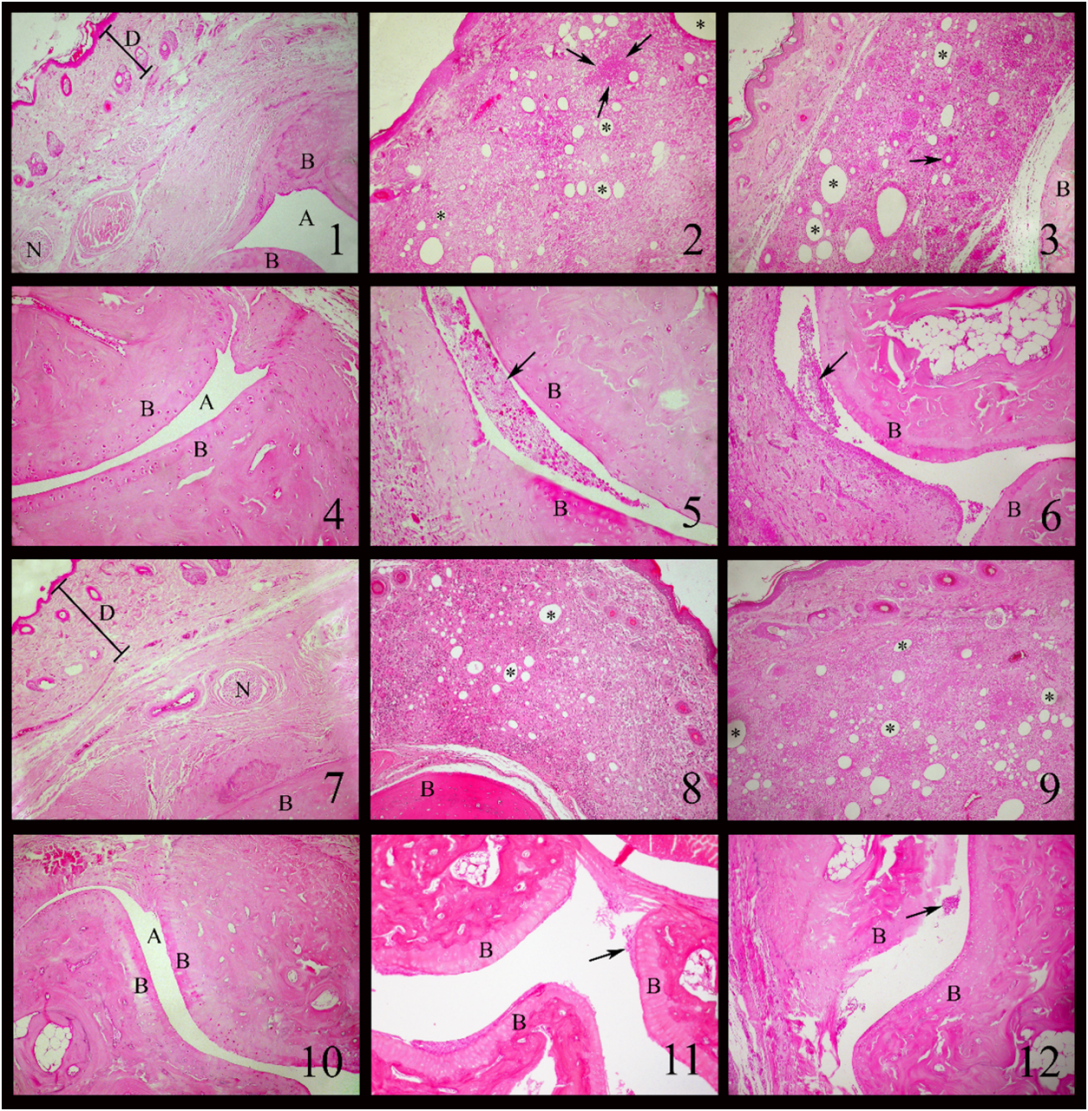
Histological profiles of the paws of rats after the CFA-induced RA (0.1 mL, i.pl.) and treatment with 4-(phenylselanyl)-2H-chromen-2-one (4-PSCO) (1 mg kg^–1^, i.g.) using HE staining: **(5.1)** young control group - Tarsal region. Dermis (D). Bone (B). Joint space (A). Nerve (N). HE. 100X. **(5.4)** young control group - Tarsal region. Bone (B). Joint space (A). HE. 100X. **(5.7)** aged control group - Tarsal region. Dermis (D). Bone (B). Nerve (N). HE. 100X. **(5.10)** aged control group - Tarsal region. Bone (B). Joint space (A). HE. 100X. **(5.2)** young CFA-induced group - Tarsal region. Dermis and subcutaneous tissue show a large amount of dilated lymphatic vessels (*). Abundant inflammatory infiltrates of eosinophils, neutrophils, and mononuclear cells. Eosinophilic infiltrates (between the arrows). HE. 100X. **(5.9)** aged CFA-induced group - Tarsal region. Subcutaneous showing a large amount of dilated lymphatic vessels (*), abundant inflammatory infiltrates of eosinophils, neutrophils, and mononuclear cells. HE. 100X. **(5.5)** young CFA-induced group - Tarsal region. Joint space showing fibrin, cell debris, and red blood cells. Bone (B). HE. 100X. **(5.6)** aged CFA-induced group - Tarsal region. Joint space showing fibrin, cell debris, and red blood cells. Bone (B). HE. 100X. **(5.8)** young CFA-induced +4-PSCO group - Tarsal region. Inflammatory response dispersed throughout the dermis and subcutaneous tissue, but less pronounced, with fewer dilated lymphatic vessels (*). Bone (B). HE. 100X. **(5.11)** young CFA-induced +4-PSCO group - Tarsal region. Joint space showing cellular debris (arrow). Bone (B). HE. 100X. **(5.3)** aged CFA-induced +4-PSCO group - Tarsal region. The inflammatory response is more restricted to the subcutaneous region with a multifocal accumulation of eosinophils and inflammatory infiltrates of mononuclear cells. A smaller amount of dilated lymphatic vessels (*). Eosinophilic perivasculitis (arrow). Bone (B). HE. 100X. **(5.12)** aged CFA-induced +4-PSCO group - Tarsal region. Joint space showing cellular debris (arrow). Bone (B). HE. 100X.

In young rats treated with 4-PSCO (1 mg kg^–1^, i.g.), there was a moderate increase in the metatarsophalangeal region compared to the young CFA-induced, aged CFA-induced, and aged CFA-induced +4-PSCO groups. Edema and inflammatory infiltrate were more discrete, and some lymphatic vessels were dilated. The foci of eosinophilic inflammatory infiltrate in the dermis and subcutaneous tissue were more discrete (Figure 7 **.8**), and the interosseous connective tissue had a scarce mononuclear inflammatory infiltrate. The intra-articular space showed a mild eosinophilic inflammatory infiltrate, and some necrotic cells were observed on the joint surfaces (Figure 5 **.11**).

In aged rats treated with 4-PSCO, a moderate increase in the metatarsophalangeal region was observed compared to the young and aged CFA-induced groups. The interosseous and subcutaneous connective tissue showed moderate histological changes. The dermis was well organized and contained a mild inflammatory mononuclear infiltrate. Slight lymphatic vessel dilatation, moderate eosinophilic inflammatory infiltrate, edema, and clear eosinophilic perivasculitis were observed (Figure 5 **.3**). The synovial membrane exhibited loss of lining epithelial cells, reduced congestion, and mild eosinophilic infiltrate. Some residual cellular debris remained in the intra-articular space (Figure 5 **.12**). In summary, 4-PSCO treatment reduced the CFA-induced damage in the paws of young and aged rats.

### 2.4 4-PSCO Reduces Mechanical and Thermal Sensitivities Induced by CFA and Aggravated by Aging

The results regarding mechanical sensitivity induced by CFA in young and aged rats are shown in Figure 6A. Aging *per se* promoted a reduction in the withdrawal threshold in the von Frey test, indicating an increased mechanical sensitivity compared with the young group. Intraplantar (i.pl.) administration of CFA further reduced the paw withdrawal threshold in young and aged animals. Thus, CFA increased mechanical sensitivity in young and aged rats. Interestingly, mechanical sensitivity in the aged CFA-induced rats was higher than that of young CFA-induced rats, as evidenced by a lower paw withdrawal threshold on day 7 (Figure 6A). On days 7 and 21 after CFA exposure, aged rats exhibited 47% reduction (ANOVA: *F*_(2, 36)_ = 292.9, *p* < 0.0001) and 55% reduction (ANOVA: *F*_(2, 36)_ = 383.5, *p* < 0.0001) in paw withdrawal threshold, respectively. Similarly, on day 21, young rats exposed to CFA showed a 53% reduction in threshold. Notably, daily administration of 4-PSCO (1 mg kg^–1^, i.g.) significantly attenuated the reduction of paw withdrawal threshold, thereby reducing mechanical sensitivity in CFA-exposed rats by 90% in young and 41% in aged animals (Figure 6A).

**Figure 6:**
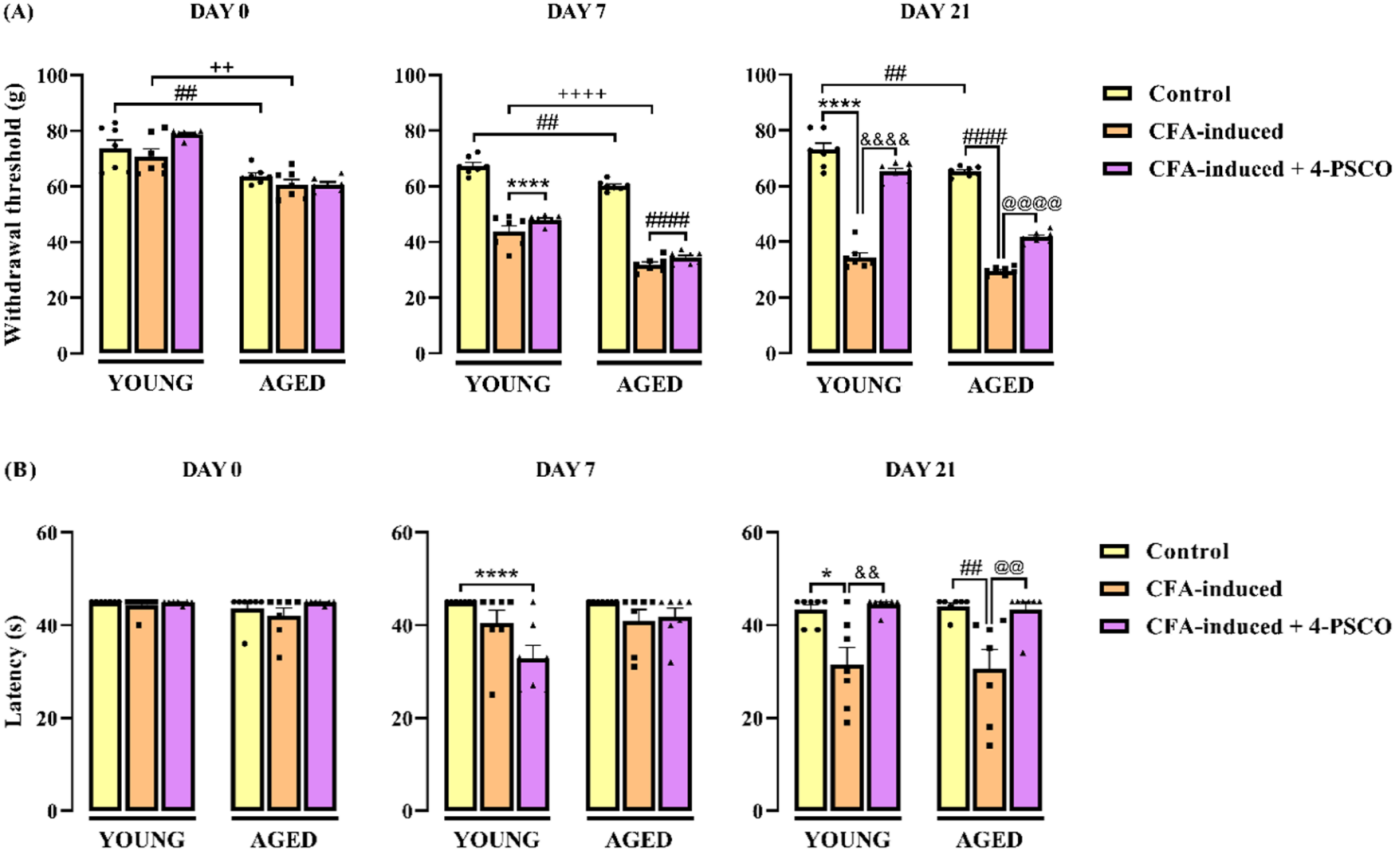
Effects of aging and 4-(phenylselanyl)-2H-chromen-2-one (4-PSCO) (1 mg kg^–1^, i.g.) on the paw withdrawal threshold for mechanical stimulus in the von Frey test (A) and on the thermal hyperalgesia in the hot plate test (B) of rats after the CFA-induced RA (0.1 mL, i.pl.). Each point represents the mean of 7 rats in each group. (*) *p* < 0.05, and (****) *p* < 0.0001 denote significance levels compared with the young control group; (##) *p* < 0.01, and (####) *p* < 0.0001 denote significance levels compared with the aged control group; (++) *p* < 0.01, and (++++) *p* < 0.0001 denote significance levels compared with the aged CFA-induced group; (&&) *p* < 0.01, and (&&&&) *p* < 0.0001 denote significance levels compared with the young CFA-induced +4-PSCO group; (@@) *p* < 0.01, and (@@@@) *p* < 0.0001 denote significance levels compared with the aged CFA-induced +4-PSCO group (Two-way ANOVA followed by Tukey’s test).

Thermal sensitivity results are presented in Figure 6B. Baseline latencies to thermal stimuli were equivalent among all experimental groups before CFA induction (Day 1). On day 21, both young and aged animals that received CFA administration showed a significant reduction in latency time, confirming that CFA increased thermal sensitivity in the animals of the young (27%) and aged (∼31%) groups compared with their respective control groups (ANOVA: *F*_(2, 36)_ = 18.59, *p* < 0.0001). However, no statistical difference was found between the young and aged CFA-induced groups. Significantly, 4-PSCO (1 mg kg^–1^, i.g.) effectively attenuated CFA-induced thermal hypersensitivity in both young (41%) and aged (42%) rats (Figure 6B).

### 2.5 Oxidative Stress Exacerbates CFA-Induced RA

To highlight the role of oxidative damage in the development of CFA-induced RA in young and aged rats, we assessed the levels of RS, thiobarbituric acid reactive species (TBARS), and nitrite and nitrate (NOx) in the spinal cord and paws of the animals. Our results demonstrated elevated levels of TBARS and RS in both the paw (Figure 7A,7C) and spinal cord (Figure 7B,7D) of young and aged CFA-induced rats compared to their respective control groups. However, aging did not significantly influence TBARS (Figure 7C) and RS (Figure 7A) levels in the paws, nor did it affect RS levels in the spinal cord (Figure 7B). In contrast, significant differences in TBARS levels in the spinal cord were observed between the young and aged CFA-induced groups (Figure 7D).

**Figure 7:**
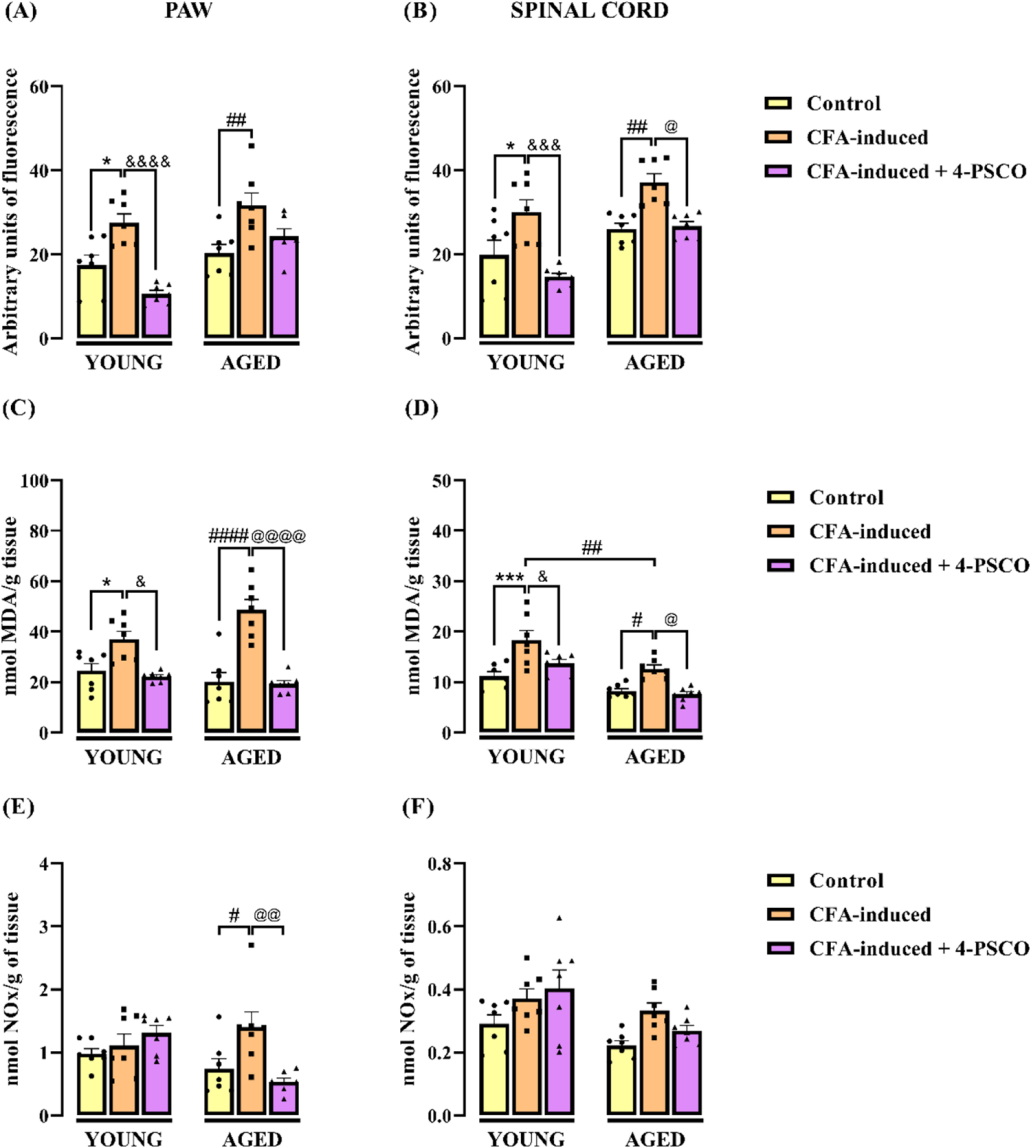
Effects of aging and 4-(phenylselanyl)-2H-chromen-2-one (4-PSCO) (1 mg kg^–1^, i.g.) on RS levels (A) in the paw and (B) spinal cord; TBARS (C) in the paw and (D) spinal cord; NOx (E) in the paw and (F) spinal cord of rats after the CFA-induced RA (0.1 mL, i.pl.). Each column represents the mean ± SEM of 7 rats in each group. (*) *p* < 0.05, and (***) *p* < 0.001 denote significance levels compared with the young control group; (#) *p* < 0.05, (##) *p* < 0.01, and (####) *p* < 0.0001 denote significance levels compared with the aged control group; (&) *p* < 0.05, (&&&) *p* < 0.001, and (&&&&) *p* < 0.0001 denote significance levels compared with the young CFA-induced +4-PSCO group; (@) *p* < 0.05, (@@) *p* < 0.01, and (@@@@) *p* < 0.0001 denote significance levels compared with the aged CFA-induced +4-PSCO group (Two-way ANOVA followed by Tukey’s test).

In this context, treatment with 4-PSCO reversed the CFA-induced increase in TBARS levels in the paw (ANOVA: *F*_(2, 36)_ = 34.50, *p* < 0.0001) (Figure 7C), as well as TBARS (*F*_(2, 36)_ = 19.64, *p* < 0.0001) (Figure 7D) and RS levels (*F*_(2, 36)_ = 20.45, *p* < 0.0001) in the spinal cord of young and aged rats compared to young, and aged CFA-induced groups, respectively. Specifically, 4-PSCO reduced RS levels in the spinal cord by 51% in young and 28% in aged CFA-induced rats, compared to their untreated groups. However, in the paw, 4-PSCO treatment was effective in reducing RS levels only in the young animals (ANOVA: *F*_(2, 36)_ = 19.60, *p* < 0.0001) (63%) (Figure 7A).

Regarding reactive nitrogen species, NOx levels in the paw and spinal cord of young CFA-induced rats were unchanged compared to the young control group (Figure 7F). In contrast, aged CFA-induced rats showed significantly higher NOx levels in the paw (89%) compared to aged control group (ANOVA: *F*_(2, 36)_ = 3.738, *p* < 0.05) (Figure 7E). No significant differences in NOx levels in the paw and spinal cord were observed between young and aged control animals, or between young and aged CFA-induced groups (Figure 7E,7F). Notably, treatment with 4-PSCO (1 mg kg^–1^, i.g.) reversed CFA-induced increase NOx levels in the paw (62%) (Figure 7E) of aged rats compared to the untreated aged CFA-induced group. However, 4-PSCO treatment did not reduce CFA-induced NOx levels in the spinal cord of aged rats (*p* > 0.05; Figure 7F).

### 2.6 The 4-PSCO Exerts Antioxidant Effects against CFA-Induced Oxidative Stress Exacerbated by Aging in Rats

The effects of 4-PSCO and/or CFA on catalase (CAT) activity and nonprotein thiol (NPSH) levels in young and aged rats are presented in Figure 8. Aged CFA-induced animals exhibited a reduction of CAT activity in the paw (54%) (Figure 8A) and in the spinal cord (43%) samples (Figure 8B) when compared to their control group. Similarly, CAT activity was reduced in the paw (∼52%) (Figure 8A) and spinal cord (57%) (Figure 8B) of young CFA-induced rats compared to their control group. Statistical analysis revealed that 4-PSCO (1 mg kg^–1^, i.g.) restored CAT activity to control levels in both paw (ANOVA: F _(2, 36)_ = 19.67, *p* < 0.0001) and spinal cord (ANOVA: F _(2, 36)_ = 14.33, *p* < 0.0001) tissues of young rats, whereas in aged animals, CAT activity was restored only in the paw. Notably, no significant differences in CAT activity in the paw or spinal cord were observed between the young control and young CFA-induced groups (Figure 8A), or between the aged control and aged CFA-induced groups (Figure 8B).

**Figure 8:**
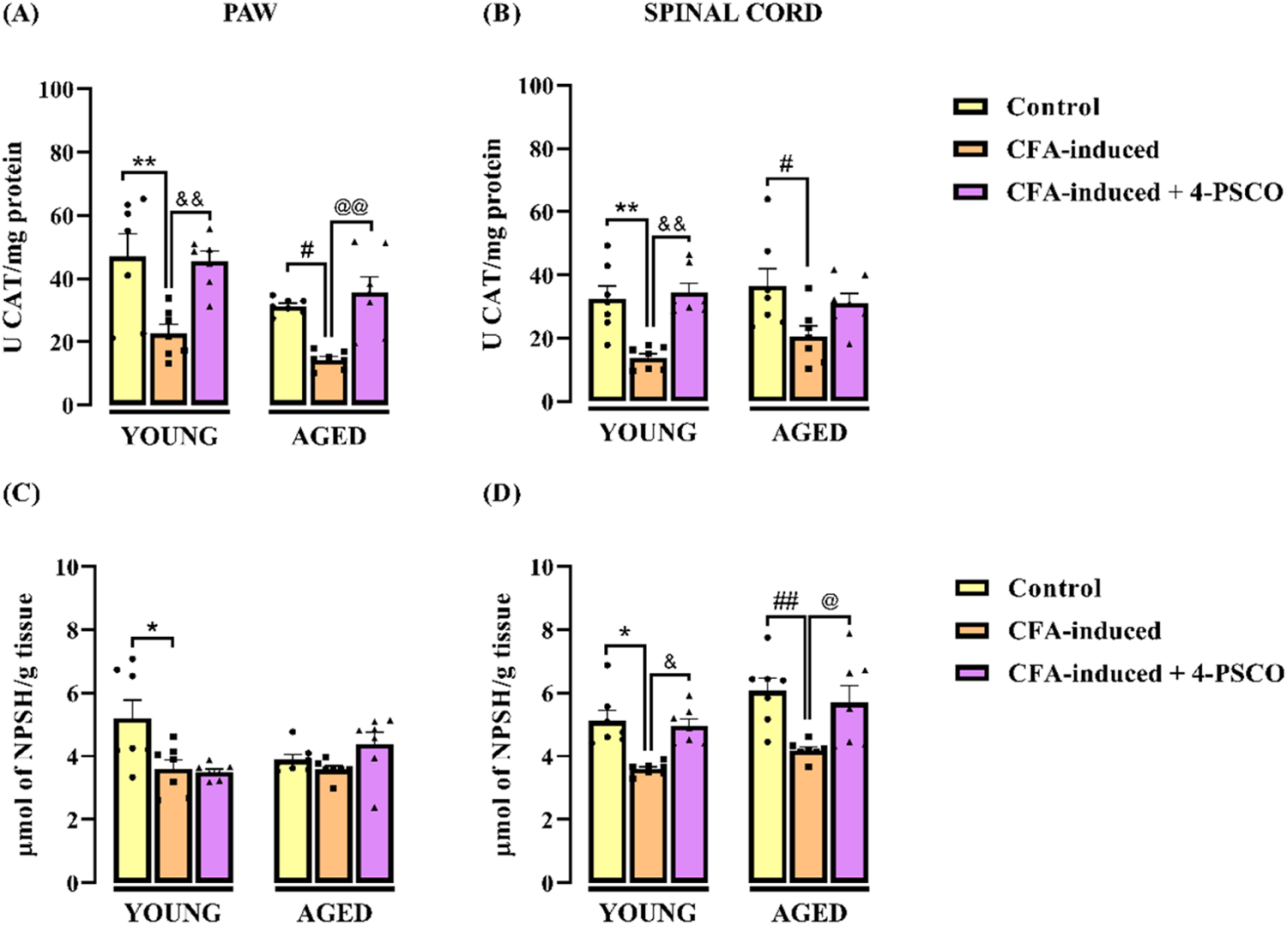
Effects of aging and 4-(phenylselanyl)-2H-chromen-2-one (4-PSCO) (1 mg kg^–1^, i.g.) on CAT activity (A) in the paw and (B) spinal cord of rats; NPSH levels (C) in the paw and (D) spinal cord of the rats after the CFA-induced RA (0.1 mL, i.pl.). Each point represents the mean of 7 rats in each group. (*) *p* < 0.05 and (**) *p* < 0.01 denote significance levels compared with the young control group; (#) *p* < 0.05 and (##) *p* < 0.01 denote significance levels compared with the aged control group; (&) *p* < 0.05 and (&&) *p* < 0.01 denote significance levels compared with the young CFA-induced +4-PSCO group; and (@) *p* < 0.05 and (@@) *p* < 0.01 denote significance levels compared with the aged CFA-induced +4-PSCO group (Two-way ANOVA followed by Tukey’s test).

Our findings showed that aged CFA-induced rats exhibited a decrease in NPSH levels in the spinal cord (Figure 8D) compared to the aged control group. However, no alteration in NPSH levels in the paws of aged rats with CFA-induced RA was observed (Figure 8C) (*p* > 0.05). Additionally, NPSH levels in the paw (ANOVA: *F*_(2, 36)_ = 4.626, *p* < 0.05) (Figure 8C) and spinal cord (ANOVA: *F*_(2, 36)_ = 16.46, *p* < 0.0001) (Figure 8D) of young rats exposed to CFA decreased compared with the young group. In the paw and spinal cord, no statistical difference was observed between the young control, the young CFA-induced, and the aged control, and the aged CFA-induced groups, respectively. Notably, 4-PSCO normalized NPSH levels in the spinal cord of young and aged CFA-exposed rats compared with the young and aged CFA-induced groups, respectively (Figure 8D).

### 2.7 Aging Modulates Spleen and Thymus Indices in CFA-Induced Arthritic Rats

Our findings revealed that the spleen index in aged CFA-induced rats was markedly increased compared with the aged control group (Figure S1A). Meanwhile, the thymus index decreased in the aged CFA-induced rats (Figure S1B) compared to the aged control group. The young CFA-induced animals also showed a change in the spleen (Figure S1A) and thymus (Figure S1B) indices compared to the young control groups. Additionally, the thymus index was not statistically different between young and aged controls, nor between young and aged CFA-induced groups. However, significant differences in the spleen index were observed between young and aged controls and between the young control and aged CFA-induced groups. Notably, the data showed that the spleen index of the 4-PSCO-treated animals was normalized compared with the young and aged CFA-induced groups (ANOVA: *F*_(2, 36)_ = 24.58, *p* < 0.0001) (Figure S1A). Nevertheless, 4-PSCO did not restore the thymus weight in the young and aged rats with CFA-induced RA (*p* > 0.05) (Figure S1B).

## 3 Discussion

RA is a disabling autoimmune disease related to cartilage and bone impairments, along with chronic joint inflammation. Pain is one of the most challenging symptoms for RA patients, and the contribution of inflammation to RA-related pain is well established.^14^ In this context, aging is also considered a complex process, and the chronic pro-inflammatory state is a pervasive condition in RA. Immunosenescence, characterized by the decline in immune competence, impaired DNA repair mechanisms, excessive release of pro-inflammatory cytokines, accumulation of senescent cells, and elevated levels of reactive oxygen species collectively underscore aging as a key factor contributing to both the development and persistence of RA.^32^ Furthermore, studies have suggested that aging itself contributes to the development of pain^33,34^ and here we demonstrate that aging exacerbates mechanical and thermal hyperalgesia induced by CFA, primarily through inflammatory process and oxidative damage to both the central and peripheral nervous systems.

A widely used preclinical model to study RA involves CFA-induced arthritis due to its pathological similarity to human condition. CFA induces polyarthritis in animals in two stages: an initial stage (days 0–10), characterized by primary lesions, followed by a chronic stage (days 11–28), typically associated with synovitis and contralateral lesions.^35^ Our study showed CFA-induced RA-like signs and symptoms in both young and aged rats, as evidenced by increased paw diameter, circumference, and edema across both age groups. Notably, aged rats induced to CFA exhibited more pronounced signs and symptoms than young animals. These findings align with previous research indicating age as an aggravating factor of RA susceptibility. While younger rats showed earlier symptoms, the progression, severity, and chronicity of arthritis differed from that observed in aged rats.^36,37^ Moreover, arthritis symptoms, including hind paw swelling, became apparent 3–4 days after CFA induction, reflecting both acute and chronic inflammation phases.^37^

In this manner, CFA injection into the paw of both young and aged rats caused persistent swelling in multiple joints, accompanied by inflammatory cells. The invasion of tissue by noxious stimuli activates a cascade of intracellular signaling pathways via the cell surface receptors on resident cells, releasing various inflammatory mediators.^6,38,39^ As expected, histological analysis confirmed that CFA-induced rats exhibited a robust inflammatory infiltrate composed of eosinophils and bleeding, edema, and dilated lymphatic vessels. These histological changes are consistent with previous findings by Zhao et al.^40^ who demonstrated that macrophage activation and infiltration at affected sites can result in significant tissue alteration, including edema and cartilage degeneration. Importantly, treatment with 4-PSCO significantly ameliorated RA-associated signs and symptoms and attenuated CFA-induced histopathological damage in the paws of both young and aged animals. These findings underscore the therapeutic potential of 4-PSCO as a novel anti-inflammatory agent capable of modulating both the progression and severity of RA-related inflammation.

Selenocoumarin compounds, such as 4-PSCO, are particularly interesting due to the strategic inclusion of a coumarin moiety within an organoselenium scaffold. While the pharmacological potential of organoselenium compounds is well-documented, our findings provide additional insight by demonstrating the added therapeutic benefit derived from incorporating the coumarin nucleus into their structure. Coumarins possess a conjugated, fused-ring system that renders them highly suitable for various pharmacological applications. Their structural simplicity, combined with the chemical reactivity of the benzene and pyrone rings, is a defining feature of this compound class.^41^ Notably, the planarity of the aromatic rings, presence of a lactone group, and ability to engage in hydrogen bonding are critical to ligand-protein interactions, thereby enhancing target recognition and pharmacodynamic effects.^42^ Furthermore, structural modifications at key positions—C-3, C-4, and C-7—have been shown to enhance the biological activity of synthetic coumarin derivatives.^43^ A growing body of evidence has highlighted the broad bioactivity profile of selenocoumarin-based compounds, particularly their interaction with multiple biological targets implicated in nociceptive and inflammatory processes.

In our study, treatment with 4-PSCO significantly suppressed MPO activity in paw tissue, underscoring the potential relevance of MPO inhibition in controlling inflammatory responses, preventing joint damage in RA, and attenuating oxidative stress-induced injury. In the RA, MPO is consistently detected at high concentrations in patients’ synovial fluid and serum and has been implicated in bone erosion and joint degradation.^44–47^ Here, we observed markedly elevated MPO activity in the paw tissue of both young and aged rats following CFA administration, reinforcing the role of MPO as a critical mediator in RA pathophysiology. MPO serves as a primary enzyme released by activated neutrophils, catalyzing the reaction of hydrogen peroxide (H_2_O_2_) to produce hypochlorous acid (HOCl) and other RS. These oxidants can induce extensive oxidative modifications to lipids, proteins, and extracellular matrix components.^48–50^ Supporting this, Prokopowicz et al.^50^ demonstrated that HOCl is a highly potent oxidizing agent capable of damaging key structural components of cartilage in RA patients.

In a previous study, our research group investigated the antiedematogenic and anti-inflammatory properties of 4-PSCO using well-established experimental models of pain and inflammation.^31^ The administration of low doses of 4-PSCO significantly reduced paw-licking behavior and edema following i.pl. injection of glutamate. These findings are consistent with those of the present study and further support the hypothesis that 4-PSCO effectively modulates the inflammatory cascade by suppressing the amplification of pro-inflammatory cytokines and chemical mediators.

CFA is known to induce chronic inflammatory pain by releasing inflammatory mediators that activate and sensitize nociceptive neurons, ultimately leading to reduced pain thresholds.^7,51,52^ This nociceptive sensitization is maintained by a robust local inflammatory response involving the infiltration of various immune cells, such as dendritic cells, activated T lymphocytes, macrophages, B cells, neutrophils, fibroblasts, and osteoclasts, as observed in the histological analyses and reflected by increased MPO activity.^17^ While CFA is widely used to model inflammation-associated pain, the mechanisms driving CFA-induced neuropathic components remain unclear. Furthermore, current pharmacological strategies for RA are focused mainly on reducing inflammation, often failing to address the complex mechanisms underlying pain adequately. In this study, young and aged CFA-induced rats exhibited a significant decrease in nociceptive thresholds, as assessed by behavioral tests (von Frey and hot plate), indicating mechanical and thermal hyperalgesia development. Our findings demonstrate that aging exacerbates CFA-induced mechanical allodynia, suggesting that age-related impairments in endogenous pain inhibitory mechanisms contribute to enhanced pain sensitivity.^33^

These results align with previous studies, including those by Koop et al.,^15^ which reported neuropathic-like symptoms in chronic polyarthritis models resembling those observed in RA patients. Furthermore, studies by Gomes et al.^53^ and Noh et al.^37^ have indicated that allodynia and hyperalgesia commonly experienced by RA patients are associated with extensive infiltration of macrophages and fibroblasts within the joints. The resulting inflammatory response sensitizes peripheral nociceptors by inducing the phosphorylation of ligand-gated channels, such as voltage-gated sodium channels, which alters membrane properties and increases the frequency of action potential firing, ultimately enhancing sensitivity to thermal and mechanical stimuli.^54–56^

In addition to voltage-gated sodium channels, other ion channels and pattern recognition receptors, such as transient receptor potential vanilloid 1 (TRPV1) and Toll-like receptors (TLRs), have been detected in the synovial fluid of patients with RA, where they contribute significantly to nociceptive signaling mediated by inflammatory stimuli.^57^ In line with this, our research group recently demonstrated that 4-PSCO exhibits high binding affinity for several molecular targets critically involved in inflammatory signaling cascades, including mitogen-activated protein kinase p38 (p38 MAPK), peptidylarginine deiminase type 4 (PAD4), phosphoinositide 3-kinase (PI3K), Janus kinase 2 (JAK2), Toll-like receptor 4 (TLR4), and nuclear factor kappa B (NF-κB).^31^

Notably, the molecular docking analyses revealed that the interactions of 4-PSCO with these targets were not only comparable to, but in some instances exceeded, those observed for reference anti-inflammatory and analgesic agents currently employed in clinical settings.^58–60^ These findings strongly suggest that 4-PSCO may exert its therapeutic effects through mechanisms akin to established pharmacological agents, particularly via modulation of key inflammatory pathways.

Moreover, it is well established that central sensitization mechanisms contribute to exacerbating hyperalgesia in RA by lowering the activation threshold of nociceptive neurons, resulting in an amplified and persistent perception of pain.^52,55^ This phenomenon is a significant factor in the chronicity and severity of RA-associated pain and substantially impacts patients’ functional capacity and quality of life. The present study offers compelling preclinical evidence that 4-PSCO may represent a promising therapeutic strategy for pain management in RA. The compound effectively attenuated mechanical and thermal hypersensitivities in a CFA-induced RA model, irrespective of age.

These findings are further supported by previous research demonstrating the acute antinociceptive effects of 4-PSCO, as evidenced by increased thermal response latency in the hot plate test and a significant reduction in mechanical hypersensitivity in mice subjected to CFA-induced inflammation, as assessed by the von Frey test.^27^ Organoselenium compounds and coumarin derivatives have been shown to modulate diverse signaling pathways involved in nociceptive processing, particularly those with supraspinal involvement.^61,62^ Altogether, these findings support the hypothesis that 4-PSCO exerts its analgesic effects, at least partially, through central mechanisms, underscoring its potential as a promising pharmacological strategy for managing pain and inflammation associated with RA.

During aging, the body’s immune system is deeply involved and undergoes progressive changes, leading to alterations to immune tissues and organs. Among these, the thymus and spleen are key organs essential for immune function. The spleen acts as a primary defense mechanism against pathogens by filtering blood and removing aged or damaged blood cells, while the thymus is responsible for the maturation of T-lymphocyte precursors into functional T-cells.^63^ In our study, aged CFA-induced rats exhibited a marked increase in spleen index and a significant reduction in thymus index. Additionally, these animals experienced notable weight loss following CFA administration. These findings are consistent with prior research suggesting a correlation between thymus and spleen indexes and decreased body weight during aging.^35,64^ Furthermore, our results demonstrate that treatment with 4-PSCO effectively reduced the spleen index in young animals and restored body weight in aged animals. These outcomes highlight the therapeutic potential of 4-PSCO in modulating immune system activity, which is particularly relevant in autoimmune and inflammatory conditions such as RA.

We evaluated potential changes in oxidative stress markers to shed light on additional mechanisms involved in CFA-induced RA. The pathophysiology of RA is highly complex, with oxidative stress playing a significant role in initiating and sustaining the pro-inflammatory environment characteristic of the disease. Growing evidence indicates that levels of RS, including superoxide and hydroxyl radicals, and lipid peroxidation, are significantly elevated in the peripheral blood and synovial tissue of RA patients.^44,45,63^ Similarly, nitric oxide metabolites (NOx) are key signaling molecules, produced during the inflammatory response by activated cells and macrophages.^38,44^ As expected, CFA induction significantly increased RS, TBARS, and NOx levels in two different tissues (paw and spinal cord) of aged rats.

Recent evidence shows that aging is closely associated with dysfunction of the antioxidant defense system and impaired immune function, which contribute to the secretion of pro-inflammatory mediators, an accumulation of senescent cells, and elevated RS production.^65–70^ In addition, the buildup of oxidized cellular components and degradation byproducts can further amplify the synovial inflammation.^68^ Studies have demonstrated that aging leads to increased oxidative damage and a decline in antioxidant capacity, exacerbating RA progression and intensifying pain sensitivity.^71,72^

In this context, antioxidant defenses are crucial in aging and inflammation. These systems tightly regulate RS inactivation and modulate the inflammatory response.^70,72,73^ Reduced glutathione, the primary nonprotein thiol measured in the NPSH assay, is essential for maintaining redox balance.^74^ However, excessive oxidative stress can overwhelm these defenses, leading to enzymatic dysfunction and tissue damage.^38,49^ In our study, CFA significantly inhibited catalase (CAT) activity in the paw and spinal cord of aged rats and reduced NPSH levels in the spinal cord. These findings are in agreement with previous studies that have reported reductions in the activity of key antioxidant enzymes, such as superoxide dismutase (SOD) and CAT, along with decreased levels in nonenzymatic antioxidants in inflamed articular tissues.^38,63,75^ This depletion of the antioxidant system reinforces the hypothesis that oxidative stress plays a central role in RA pathogenesis by contributing to cellular damage and amplifying the inflammatory process.

Importantly, our findings demonstrate for the first time that 4-PSCO attenuated oxidative damage by normalized CAT activity in the paw and spinal cord, restored NPSH levels in the spinal cord, and reduced pain sensitivity in young and aged CFA-induced rats. These results underscore the promising pharmacological potential of 4-PSCO in modulating oxidative stress and inflammation, independent of age.

In summary, this study highlights the multifaceted effects of 4-PSCO and its involvement in multiple interconnected biological pathways. Our findings demonstrate that CFA-induced rheumatoid arthritis exacerbates pain sensitivity through mechanisms associated with oxidative damage. Remarkably, 4-PSCO treatment effectively reversed both mechanical and thermal allodynia, attenuated the inflammatory response, and reduced oxidative stress. Importantly, few studies have addressed pain as a central and urgent clinical issue in RA management, particularly in elderly populations, where pain is often more pronounced and debilitating. Altogether, our results position 4-PSCO as a promising therapeutic candidate for developing safer and more effective strategies for RA treatment, while also emphasizing the relevance of age-related differences in the pathophysiology and progression of the disease.

## 4 Experimental Section

### 4.1 Material and Methods

#### 4.1.1 Animals and Ethical Approval

The experiments were carried out using young (2-month-old, weighing 300–500 g) and aged (24-month-old, weighing 500–700 g) male Wistar rats. The animals were obtained from a local breeding colony, housed in cages with access to food and water *ad libitum*, and kept in a separate air-conditioned (22 ± 2 °C) room on a 12-h light/dark cycle with lights on at 7:00 am. The experimental protocols were authorized by the Committee on Care and Use of Experimental Resources of the Federal University of Pelotas (Brazil), affiliated with the National Council for the Control of Animal Experimentation, and registered under CEUA no. 24440-2019, 014903/2022-40. Animal care and behavioral assays complied with the National Institutes of Health Guide for the Care and Use of Laboratory Animals (NIH Publications no. 80–23, revised in 1996) and the International Guiding Principles for Biomedical Research Involving Animals.^76^ All efforts were made to minimize the number of animals used and their suffering, and all experiments were performed between 8:00 am and 5:00 pm.

#### 4.1.2 Chemicals

Complete Freund’s adjuvant (CFA) (Santa Cruz Biotech., EUA) was obtained from Sigma-Aldrich Chemical Co. (St. Louis, MO, USA). The chemicals used in this study were of analytical grade and obtained from standard commercial suppliers. The 4-PSCO (Figure 9) was synthesized and characterized at the Department of Chemistry at the Federal University of Santa Maria.^29^ GC/MS analysis determined the chemical purity of this compound (99.9%). The 4-PSCO was dissolved in canola oil and administered intragastrically (i.g.) at a constant 1 mL kg^–1^ body weight. The CFA was administered intraplantar (i.pl.).

**Figure 9:**
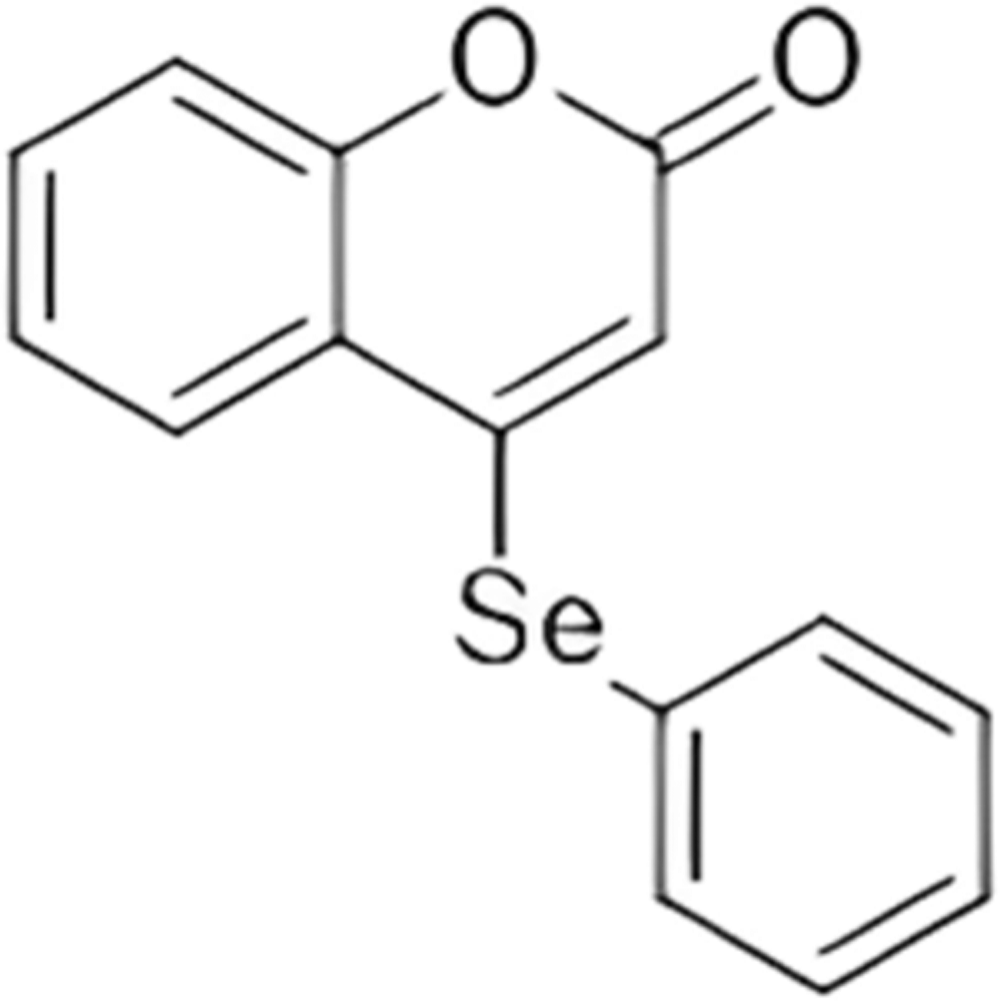
Chemical structure of 4-(phenylselanyl)-2H-chromen-2-one (4-PSCO).

#### 4.1.3 Experimental Design

##### 4.1.3.1 CFA-Induced Arthritis

The rats were randomly divided into six groups (n = 7 animals/group): (**I)** young control, (**II)** young CFA-induced, (**III)** young CFA-induced +4-PSCO, (**IV)** aged control, (**V)** aged CFA-induced, and (**VI)** aged CFA-induced +4-PSCO. Experimental arthritis was induced by CFA injection (0.1 mL, i.pl.) containing 10 mg/mL of heat-killed *Mycobacterium tuberculosis* into the rats’ left hind paw plantar surface. The young CFA-induced, aged CFA-induced, young CFA-induced +4-PSCO, and aged CFA-induced +4-PSCO rats received an injection of CFA (0.1 mL, i.pl.) in the left hind paw, while the young and aged control groups received 0.9% saline solution in the same volume and administration route, on day 1. On day 12 of the experimental protocol, the animals of the young control, young CFA-induced, aged control, and aged CFA-induced groups received canola oil (10 mL kg^–1^, i.g.). In contrast, the young CFA-induced +4-PSCO and aged CFA-induced +4-PSCO group received 4-PSCO (1 mg kg^–1^, i.g.) until day 21.^77^ The selected dose (1 mg kg^–1^) is based on previous studies that demonstrated the pharmacological effects of organoselenium compounds, encompassing antioxidant and anti-inflammatory properties.^22,78^

On days 1, 7, and 21 of the experimental protocol, the development of mechanical and thermal sensitivities, paw edema, and arthritic scores were evaluated. Immediately after the behavioral tests, the animals were euthanized by isoflurane inhalation. Paw and spinal cord samples were rapidly dissected, weighed, and stored at −20 °C for *ex vivo* assays (Figure 10).

**Figure 10:**
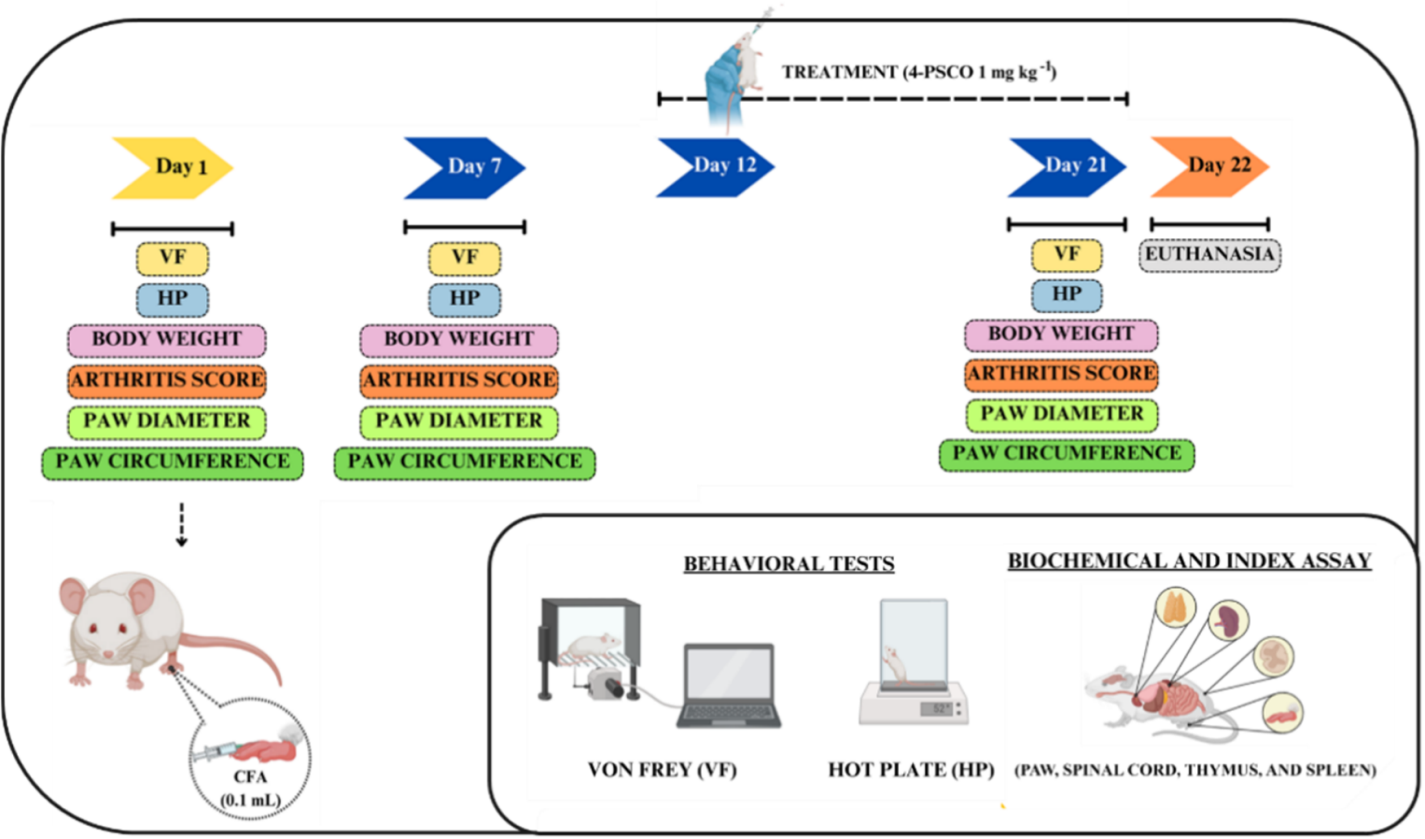
Summary of the experimental protocol.

##### 4.1.3.2 Clinical Symptoms of RA

**4.1.3.2.1. Arthritis Score:** The clinical severity of polyarthritis was determined using a visual scoring scale (0–4) according to the method described by Zhang et al.^77^ on days 0, 7, and 21. The presence of edema and erythema was noted in the ipsilateral paw. The four scoring criteria were: (0) no swelling, (1) swelling of the toe joints, (2) swelling of the toes and toe joints, (3) swelling of the ankle joints, and (4) swelling of the entire paw, which led to immobility.

**4.1.3.2.2. Evaluation of Polyarthritis and Weight Variations:** The assessment of arthritis development and establishment of the inflammatory process was evaluated on days 0, 7, and 21 after CFA induction. The joint diameter (mm) was recorded with a Vernier caliper, and the paw circumference (cm) was evaluated by wrapping a string around the paw and measuring its length on a ruler.^79^ The weight of all animals was measured on days 0, 7, 12, 16, and 21.

##### 4.1.3.3 Behavioral Tests

**4.1.3.3.1. Pain-Related Behavioral Tests**

**4.1.3.3.1.1. Mechanical Sensitivity Measurements:** Mechanical sensitivity was estimated using a digital analgesimeter (Insight, Ribeirão Preto, SP, Brazil) according to the method of Alamri et al.,^80^ with some modifications. The animals were first individually placed inside acrylic cages with wire grid floors 30 min before the start of testing in a quiet room for acclimatization. Posteriorly, the test involved evoking a hind paw flexion reflex using the digital analgesimeter adapted with a polypropylene tip. The paw withdrawal threshold was measured by applying the polypropylene tip perpendicular to the middle of the plantar surface of the hind paw at constant progressive pressure until paw withdrawal. Mechanical sensitivity was assessed at baseline (day 1) and during the experimental protocol (days 7 and 21). Data were expressed as withdrawal threshold (g).

**4.1.3.3.1.2. Thermal Sensitivity Measurements:** The thermal sensitivity was evaluated on days 0 (baseline), 7, and 21 of the experimental protocol. The rats were placed in a glass box on a heated metal plate maintained at 52 ± 1 °C, and the latency of nociceptive responses such as licking or shaking one of the paws or jumping was recorded as the reaction.^81^ Standing on the plate was limited to 45 s to avoid damage to the animal’s paws.

##### 4.1.3.4 *Ex Vivo* Assays

**4.1.3.4.1. Tissue Processing for Biochemical Analyses:** Paw samples were collected to determine paw edema, MPO activity, and histological analysis. Furthermore, oxidative stress markers, such as RS and TBARS levels, NPSH content, and CAT activity, were evaluated in the paw and spinal cord. In this sense, the oxidative stress parameters were analyzed by homogenizing the tissues in 50 mmol L^–1^ Tris HCl pH 7.4 (1/10 weight/volume) and centrifuging at 3000 rpm for 10 min to obtain a supernatant (S_1_). NOx content was also evaluated in the paw and spinal cord. The samples were homogenized in ZnSO_4_ (200 mM) and acetonitrile (96%). The homogenates were centrifuged at 14,000 rpm for 30 min at 4 °C, and the supernatant was collected for the NOx assay.

To measure MPO activity, the paw fraction was minced, pooled, and homogenized in phosphate-buffered saline (PBS; 20 mmol/L, pH 7.4) containing ethylenediaminetetraacetic acid (0.1 mmol/L). The homogenates were centrifuged at 2000 rpm for 10 min at 4 °C (fraction S_1_). Then, the S_1_ fraction was centrifuged again at 14,000 rpm at 4 °C for 15 min to yield a final pellet (P_2_) that was resuspended in a medium containing potassium phosphate buffer (50 mmol/L, pH 6.0) and hexadecyltrimethylammonium bromide (0.5 wt/v-%). The samples were frozen and thawed three times before the posterior enzymatic assay.

**4.1.3.4.2. Inflammatory Parameters**

**4.1.3.4.2.1. Paw Edema**: Paw edema was evaluated on day 21 of the experimental protocol. for this, the rats were euthanized, and both paws were amputated. The weight difference between the samples of the control paw (left) and the CFA-treated paw (right) was obtained using an analytical balance. The results obtained were represented in weight (g).

**4.1.3.4.2.2. MPO Assay:** MPO activity was assayed according to a method described elsewhere, with some modifications.^82^ In this assay, an aliquot of resuspended P_2_ (100 μL) was added to a medium containing the resuspension medium and N, N, N′, N′-tetramethylbenzidine (1.5 mmol/L). The MPO kinetic analysis was started after adding hydrogen peroxide (H_2_O_2_) (0.01% v/v), and the color reaction was spectrophotometrically measured at 655 nm at 37 °C. The results were expressed as optical density/mg protein/min.

**4.1.3.4.2.3. Histological Analysis:** The hind paws were collected and fixed by immersion in a 10% buffered formalin solution for 24 h. Subsequently, paws were decalcified in an 8% hydrochloric acid and formic acid (1:1) solution for about 5 days. Samples were cross-sectioned and routinely processed, embedded in paraffin, cut into 3–4 μm sections, stained with hematoxylin-eosin (HE), and examined under an optical microscope.

**4.1.3.4.2.4. Thymus and Spleen Indexes:** On the last day of the experimental protocol (day 22), the rats were euthanized by isoflurane inhalation, and the thymus and spleen were promptly removed and weighed. The thymus and spleen indexes were expressed as the ratio of thymus and spleen wet weight versus body weight (mg/g), respectively.^83^

**4.1.3.4.3. Oxidative Stress Parameters**

**4.1.3.4.3.1. RS levels:** The RS levels were determined according to Loetchutinat et al.^84^ For the assay, S_1_ (50 μL) was incubated with 20 μL of 2′,7′-dichlorofluorescein diacetate (DCHF-DA) (1 mmol L^–1^) and 2430 μL of Tris HCl (10 mmol L^–1^) at pH 7.4. The oxidation of DCHF-DA to fluorescent dichlorofluorescein (DCF) was measured to detect intracellular RS levels. The DCF fluorescence intensity emission was recorded at 525 nm (with 488 nm excitation) 60 min after adding DCHF-DA to the medium (Shimadzu RF-5301PC fluorometer). The RS levels were expressed as arbitrary units of fluorescence.

**4.1.3.4.3.2. TBARS Levels:** The TBARS levels were determined according to Ohkawa et al.^85^ An aliquot (200 μL) of S_1_ was incubated with 0.8% thiobarbituric acid, acetic acid buffer pH 3.4, and 8.1% sodium dodecyl sulfate at 95 °C for 2 h. The calculation of TBARS levels was initially performed by multiplying the sample absorbance by the calibration factor of the previously established malondialdehyde (MDA) curve, expressed in nmol (12.43), and dividing the result by the volume of tissue pipetted (mL) (X1). The value obtained from this initial (X1) calculation was then multiplied by it was then multiplied by the value obtained in the eq (1 divided by the protein concentration of the sample) (X2). The color reaction was measured at 532 nm. TBARS levels were expressed as nmol MDA/mg protein.

**4.1.3.4.3.3. NOx Content:** The Greiss reagent method was used to estimate tissue nitrite content.^86^ Briefly, the NOx content was estimated in a medium containing 2% vanadium chloride (in 5% HCl), 0.1% N-(1-naphthyl) ethylenediamine dihydrochloride, and 2% sulfanilamide (in 5% HCl). After incubation at 37 °C for 1 h, the color reaction was measured spectrophotometrically at 540 nm. The NOx content was calculated by multiplying the sample absorbance by the previously established average calibration factor (0.124) and dividing the result by the pipetted tissue volume (mL) and the weighed tissue amount. The concentration of nitrite/nitrate in the supernatant was determined from a sodium nitrite standard curve and expressed as μmol NOx/g of tissue.

**4.1.3.4.3.4. NPSH Content:** The NPSH content was determined using Ellman’s method.^87^ A sample of S_1_ was mixed (1:1) with 10% trichloroacetic acid (TCA). After centrifugation (3000 rpm for 10 min), the protein pellet was discarded, and free-thiol groups were determined in the clear supernatant. An aliquot of supernatant (200 μL) was added in 1 M potassium phosphate buffer pH 7.4 and 10 mM 5,5′-dithiobis-2-nitrobenzoic acid (DTNB). NPSH levels were calculated by multiplying the sample absorbance by the previously established average calibration factor (79.5) and dividing the result by the pipetted tissue volume (mL). The color reaction was measured at 412 nm and NPSH levels were expressed as nmol of NPSH/g tissue.

**4.1.3.4.3.5. CAT Activity:** The CAT activity was determined according to the method of Aebi.^88^ An aliquot of S_1_ (100 μL) and the substrate (H_2_O_2_, 105 μL) were added to a concentration of 0.3 mM in a medium containing 50 mM potassium phosphate buffer, pH 7.0. The reaction was incubated for 2 min, with kinetic measurements recorded every 30 s using a spectrophotometer at 240 nm. CAT activity was calculated based on the rate of H_2_O_2_ decomposition, which is directly proportional to the reduction in absorbance. The calculation was performed in multiple steps. The delta value was initially determined by subtracting the absorbance at 2 min from the initial absorbance. This delta value was then multiplied by the correction factor (4.6) and the previously established tissue dilution factor (X1). Subsequently, the protein concentration of the sample was determined, and this value was multiplied by the pipetted volume (mL) (X2). Finally, the result from the first calculation (X1) was divided by the second (X2) and then divided by 2. The enzymatic activity was expressed as U CAT/mg protein (1 U decomposes 1 μmol of H_2_O_2_ per minute at pH 7.0 and 25 °C).

**4.1.3.4.3.6. Protein Determination:** The protein concentration was measured according to Bradford^89^ and using bovine serum albumin as the standard. The reaction mixture contained S_1_ (50 μL) and Coomassie brilliant blue (2.5 mL) and was incubated for 10 min. The protein concentration of the sample was determined by multiplying the sample absorbance by the average calibration factor (obtained from the albumin standard curve) and the previously established tissue dilution factor. This value was then divided by the pipetted volume (mL), with protein concentration was measured spectrophotometrically at 595 nm and expressed as mg protein/mL.

##### 4.1.3.5 Statistical Analysis

All experimental findings are presented as the mean ± standard error of the mean (SEM). Statistical analysis was performed using the GraphPad Prism 6.0 software (San Diego, CA, USA). The normality of the data was evaluated using the D’Agostino and Pearson omnibus normality test. Data were analyzed using two-way ANOVA followed by Tukey’s test when appropriate. Probability values below 0.05 (*p* < 0.05) were considered statistically significant.

## 5. Limitations

Among the limitations of this study, it is important to highlight the inability to assess sex-related differences in RA outcomes, as well as the influence of aging on disease progression. Additionally, due to methodological and resource constraints, it was not feasible to conduct molecular analyses, which limited a more in-depth characterization of the underlying mechanisms involved. Another relevant limitation was the absence of a positive control group, which could not be included due to financial constraints and adherence to the ethical principles of the 3Rs (Replacement, Reduction, and Refinement), and the impossibility to conduct pharmacokinetic studies to assess the compound’s bioavailability and metabolic profile.

## 6 Perspectives

As future perspectives, and contingent upon additional funding, we aim to investigate specific intracellular signaling pathways, with a focus on Toll-like receptor 4, transient receptor potential vanilloid 1, mitogen-activated protein kinases, Janus kinase, and the transcription factor NF-κB. Moreover, we intend to evaluate the expression of voltage-gated sodium channels using qRT-PCR, considering their central role in the initiation and progression of RA. These molecular targets represent promising therapeutic avenues for delaying disease progression, modulating the inflammatory response, and alleviating chronic pain associated with RA.

## Supplementary Material



## Notes

Effects of aging and 4-(Phenylselanyl)-2H-chromen-2-one (4-PSCO) treatment on spleen and thymus indices in rats with CFA-induced RA (PDF)

## Abbreviations

RA: rheumatoid arthritis
4-PSCO: 4-(phenylselanyl)-2H-chromen-2-one
CFA: complete Freund’s adjuvant
i.pl.: intraplantar
i.g.: intragastric
MPO: myeloperoxidase
RS: reactive species
TBARS: thiobarbituric acid reactive species
NPSH: nonprotein thiol
CAT: catalase
S_1_: supernatant
PBS: phosphate-buffered saline
NOx: nitrite and nitrate
P_2_: pellet
HE: hematoxylin-eosin
DCHF-DA: 2′,7′-dichlorofluorescein diacetate
DCF: dichlorofluorescein
TCA: trichloroacetic acid
DTNB: 5,5′-dithiobis-2-nitrobenzoic acid
