# Supplementary material for: Aging is a Risk Factor for Rheumatoid Arthritis in Rats: Therapeutic Potential of 4‑(Phenylselanyl)-2H-chromen-2-one
Source: ACS Omega. 2025 Jun 9;10(24):25990–6005. doi: 10.1021/acsomega.5c02655 (PMC12198990; doi:10.1021/acsomega.5c02655)
Supplement: Supplementary file 1 [file ao5c02655_si_001.pdf]

SUPPORTING INFORMATION

Aging is a Risk Factor for Rheumatoid Arthritis in Rats: Therapeutic Potential of 4-(Phenylselanyl)-2H-chromen-2-one

Caren Aline Ramson da Fonseca[a], Jaini Janke Paltian[a], Ketlyn Pereira da Motta[a], Carolina Cristóvão Martins[a], Jean Carlo Kazmierczak[b], Ricardo Frederico Schumacher[b], Mauro Pereira Soares[c], Cristiane Luchese[a], and Ethel Antunes Wilhelm[a*]

[a]Center of Chemical, Pharmaceutical and Food Sciences, Graduate Program in Biochemistry and Bioprospecting, Federal University of Pelotas, Pelotas City, 96010-900, Brazil. [b]Chemistry Department, Graduate Program in Chemistry, Federal University of Santa Maria, Santa Maria City, 97105-900, Brazil. [c]Regional Diagnostic Laboratory, Faculty of Veterinary, Federal University of Pelotas, Pelotas City, 96010-900, Brazil.

*Address for correspondence:

Ethel Antunes Wilhelm; e-mail: ethelwilhelm@yahoo.com.br/phone: +55-53-32757360

Graduate Program in Biochemistry and Bioprospecting (PPGBBio), Center of Chemical, Pharmaceutical and Food Sciences, Federal University of Pelotas, Capão do Leão Campus, Pelotas, CEP 96010-900, RS, Brazil

SUPPLEMENTARY FIGURES

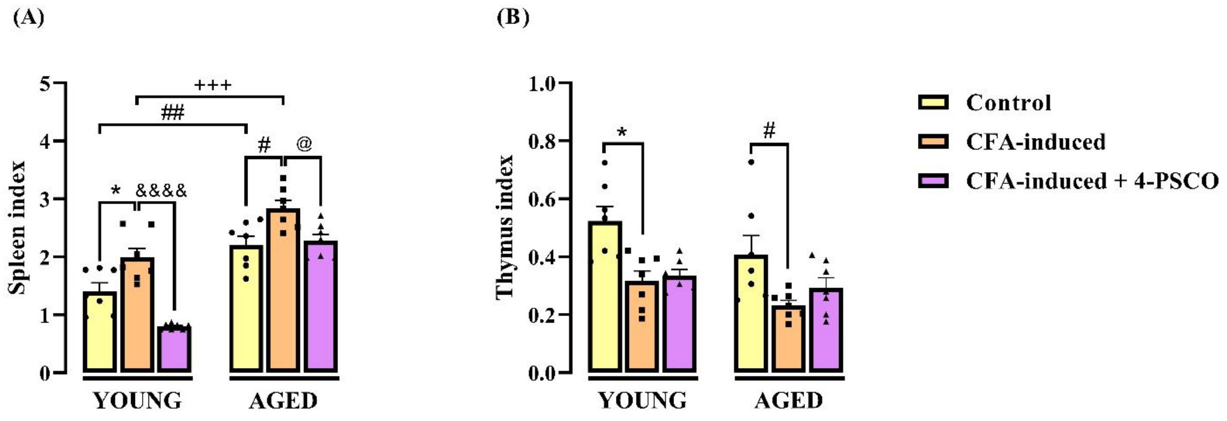

Figure S1. Effects of aging and 4-(phenylselanyl)-2H-chromen-2-one (4-PSCO) (1 mg kg^{-1}, i.g.) on the **(A)** spleen and **(B)** thymus indexes of rats after the CFA-induced RA (0.1 mL, i.pl.). Each point represents the mean of 7 rats in each group. (*) $p < 0.05$ denotes significance levels compared with the young control group; (#) $p < 0.05$, and (##) $p < 0.01$ denote significance levels compared with the aged control group; (+++) $p < 0.001$ denotes significance levels compared with the aged CFA-induced group; (&&&&) $p < 0.0001$ denotes significance levels compared with the young CFA-induced + 4-PSCO group; (@) $p < 0.05$ denote significance levels compared with the aged CFA-induced 4-PSCO group (Two-way ANOVA followed by Tukey's test).
